# Supplementary material for: Asymmetric Synthesis of Quaternary Hydantoins via a Palladium-Catalyzed Aza-Heck Cyclization
Source: J Am Chem Soc. 2025 Nov 14;147(49):44692–8. doi: 10.1021/jacs.5c16022 (PMC12703750; doi:10.1021/jacs.5c16022)
Supplement: Supplementary file 2 [file ja5c16022_si_002.zip › All NMR FID Files/S9/S9_ProtonNMR/1/pdata/1/email_EM02-264_1_1.pdf]

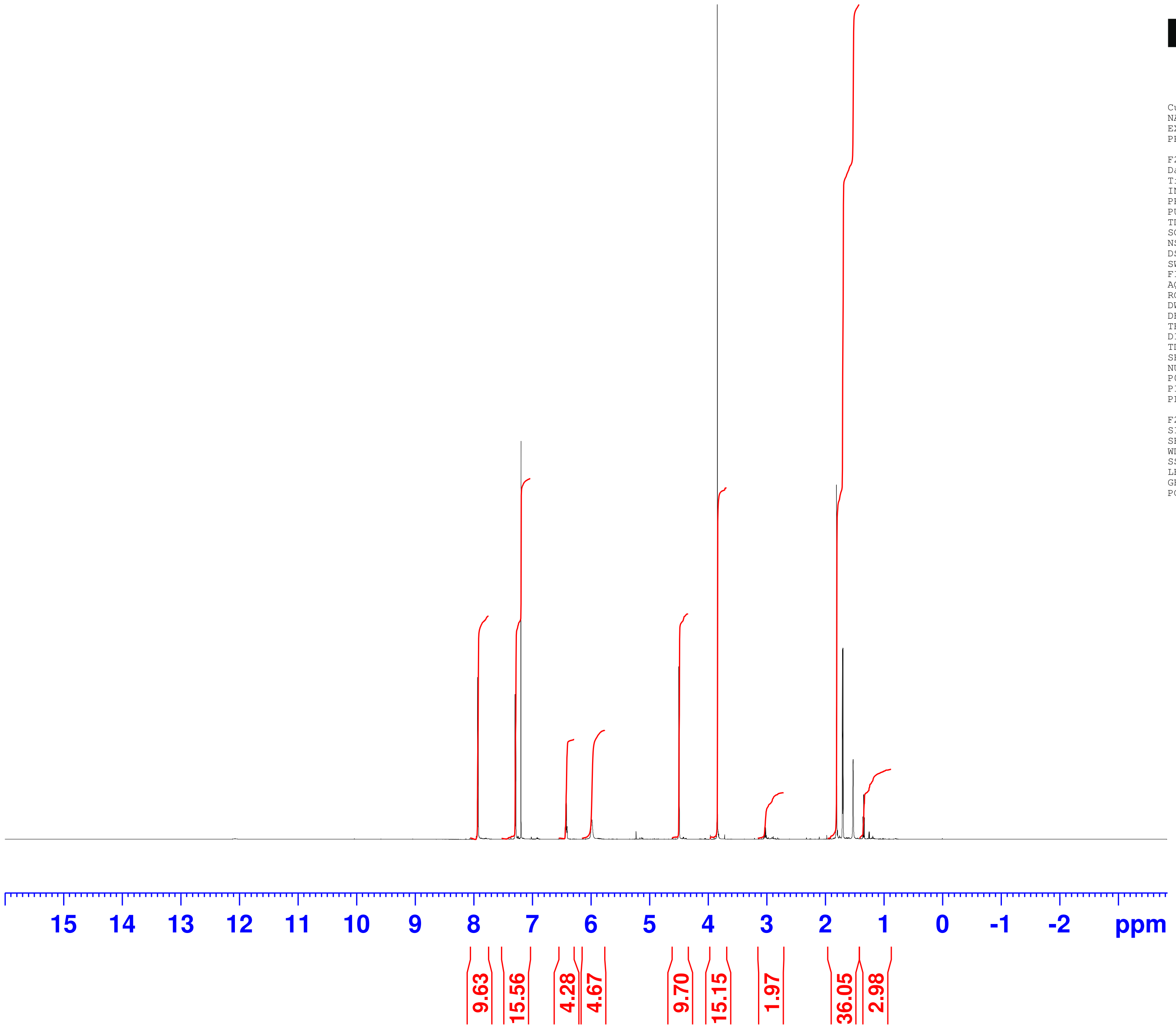


```
Current Data Parameters
NAME              EM02-264
EXPNO                    1
PROCNO                   1

F2 - Acquisition Parameters
Date_             20220624
Time                 12.50 h
INSTRUM  Avance Neo 600 at University of Delaware
PROBHD   Z185170_0001 (
PULPROG               zg30
TD                   65536
SOLVENT              CDCl3
NS                      16
DS                       2
SWH              11904.762 Hz
FIDRES            0.363304 Hz
AQ               2.7525120 sec
RG                 19.8543
DW                  42.000 usec
DE                   14.42 usec
TE                   298.0 K
D1              1.00000000 sec
TD0                      1
SFO1           600.1337058 MHz
NUC1                    1H
P0                    2.60 usec
P1                    7.81 usec
PLW1            6.89699984 W

F2 - Processing parameters
SI                   65536
SF             600.1300555 MHz
WDW                     EM
SSB                      0
LB                    0.30 Hz
GB                       0
PC                    1.00
```
